# Gender-based support systems influencing female students to pursue a bachelor of medicine, bachelor of surgery (MBBS) in Rwanda

**DOI:** 10.1186/s12909-024-05613-w

**Published:** 2024-06-07

**Authors:** Kara L. Neil, Deborah Umucyo, Agnes Binagwaho, Tsion Yohannes Waka

**Affiliations:** 1 King Faisal Hospital Rwanda, Kigali, Rwanda; 2 University of Global Health Equity, Kigali, Rwanda

**Keywords:** Gender, Inclusion, Medical education, Support systems, Mbbs

## Abstract

**Background:**

While Sub-Saharan Africa contains nearly one third of the global burden of disease, it only contains 3.5% of the healthcare workforce. Furthermore, female medical doctors are underrepresented across the continent. Studies show that increasing gender representation in medicine not only bridges this gap but may have a positive impact on patient care. This study explores the support systems influencing female students to pursue medical school in Rwanda, aiming to recommend ways to increase female participation through support systems.

**Methods:**

This is an exploratory, interpretive study employing qualitative methods. The study was conducted at thirteen secondary schools within two provinces and three universities in Rwanda that offer a medical degree program. Participants were divided into focus groups, including female and male secondary students in science and non-science combinations; teachers of secondary students; female and male students enrolled in medical school; and parents of secondary students in science and non-science combinations. Private and public, mixed and girls-only secondary schools that met the criteria were selected in each province, and all universities offering a medical degree. Participants were selected via random stratified sampling. Thirty-four semi-structured focus group discussions were conducted (28 secondary-level and 6 university-level) and 16 interviews. Data was coded inductively, with common themes identified.

**Results:**

Four main themes were identified as support systems that can either serve as facilitators or barriers to pursuing an MBBS, including teacher support, parental or familial support, financial or institutional policy support, and having access to female mentors or role models.

**Conclusion:**

Social support systems are enablers encouraging female students to join medical school. Integrating social support systems in schools and the community has the potential to increase female applicants to medical school in Rwanda.

## Background

Sub-Saharan Africa (SSA) contains 12% of the world’s population and 27% of the burden of disease, but only 3.5% of the healthcare workforce. Broadly, this overall lack of qualified health care professionals in SSA has received significant attention in recent years [1]. However, the gender disparity both in medical education and professional practice across SSA has been given far less attention yet appears to be key to the development and growth of the healthcare workforce in SSA. This gender disparity is also exemplified in Rwanda, where in 2018 the female-to-male ratio of surgical residents in Rwanda was 1 to 15.5, and the female-to-male ratio of medical students was 1 to 2.3 [2].

According to global figures, of the 234 million workers across the globe, women consist of 70% [3]. However, the data also shows that men are more likely to be in full-time employment as compared to women, and in higher ranking positions. In addition, there is occupational segregation by gender, as evidenced by the larger number of male physicians, dentists, and pharmacists, while females are more represented in nursing and midwifery. Globally from 2000 to 2017, the percentage of female physicians increased by 13% in the Organization for Economic Cooperation and Development (OECD) countries, which have the world’s largest economies [4]. However, in the case of Africa, by 2017 women still consisted of only 28% of physicians, while comprising of 65% of nurses, indicating the existence of occupational segregation and disparity.

### Gender-based support systems in medical education

Studies have shown that a lack of surrounding support systems prevent equitable gender representation in medical school, including institutional, cultural, and personal barriers [5]. From an institutional perspective, the institutional theory approach posits that gender inequities may be due to the systems and policies within the institutions themselves [6]. However, medical schools can take specific actions to increase gender equity, particularly to support women in balancing their career and family obligations without jeopardizing their educational opportunities [7]. Meanwhile, societal role expectations also contribute to support systems in place for females to pursue medicine. Role theory argues that the way we behave is framed by what our families and communities expect of us [8]. For example, one study revealed that 70% of female doctors had been discouraged to pursue surgery due to assumptions about their lack of suitability in the field [7]. While medicine is traditionally framed as a male dominated career that is unsuitable for women, having support systems that challenge these role identities may enable women to join the field [6].

While overarching theories and studies point to gender-based support systems to increase female access to medical school, these support systems should also be contextually relevant and adapted to the needs of the local context. Ultimately, “what gets measured gets done,” meaning that when contexts and institutions measure and enable transparent systems and policies to hold themselves accountable for gender equity in medicine, this is when we will make tangible progress [9].

### Gender and education in Rwanda

In the case of Rwanda, literature from the Gender Monitoring Office (GMO) indicates that Rwanda has taken significant strides towards economic inclusiveness with priority to women’s engagement in the agriculture, finance, business, communication and information sectors [9]. In 2020, there were 67,896 people engaged in the human health and social work activities in Rwanda, of whom 58% were women [10]. Detailed information on the percentage of female and male medical doctors and other health professional roles also show that women are underrepresented as health sector staff, especially as medical doctors and hospital directors, while they are overrepresented as nurses [9]. The Government of Rwanda (GOR) has developed numerous policies to promote gender equity in education, which is seen as an integrated effort between various ministries.

The vision of the *National Gender Policy* is *“*to set the Rwandan society free from all forms of gender-based discrimination and see both men and women take part fully and enjoy equitably from the development processes” [11]. Specifically, the policy provides an overview and background of gender equality issues in Rwanda, including colonial rule, the shift from subsistence to monetary economy, and the various roles of women in economic activities [11]. One of the key strategies of this policy, which has impacted both the *Education Sector Policy* and the *Girls’ Education Policy*, is to “develop gender capacity building programs for policy makers, planners, strategic and operational managers for them to acquire the right knowledge, skills, and attitudes for gender mainstreaming at all levels” [11].

Furthermore, the Ministry of Education’s (MINEDUC) *Girls’ Education Policy* aims to identify and implement strategies to promote girls’ enrollment across all levels of education, with one key strategy being to develop policies that address factors preventing girls from attending school and integrate them in national, district, and community programs and plans [12]. The six years that comprise the secondary school education in Rwanda are split between Ordinary-Level and Advanced-Level respectively, with ‘combinations’ being selected at the beginning of Advanced-Levels. With regards to girls in the sciences, a MINEDUC school census included in the 2022 *Statistical Yearbook by the National Institute of Statistics of Rwanda* (NISR) indicates that from 2017 to 2020/21, female students were the majority in science combinations (e.g., Math-Physics, Biology-Chemistry, etc.). With a female to male ratio of 1.09:1, the female majority in secondary school science combinations indicates a gender parity in this area [12].

While the *Girls’ Education Policy* does broadly identify some potential factors preventing girls from attending school, including possible family favoritism for boys and other socioeconomic factors, it does not specifically address factors contextually specific to Rwanda that may be preventing girls from applying for, or later pursuing, a Bachelor of Medicine, Bachelor of Surgery (MBBS). Having this information, however, could allow for targeted interventions to address existing gender gaps in medical education and ultimately medical practice.

### Pursuing medicine in Rwanda

In Rwanda, students aiming to become medical doctors are required to earn their Bachelor of Medicine, Bachelor of Surgery (MBBS), an academic program that is followed by an internship. Upon earning their MBBS degrees, graduates are eligible to register with the Rwanda Medical and Dental Council (RMDC) as general practitioners (GP) and later select to pursue specializations further along in their medical careers. There are currently three accredited medical schools in Rwanda, the University of Rwanda School of Medicine and Pharmacy (UR SOMP), the Adventist University of Central Africa (AUCA) and the University of Global Health Equity (UGHE). Given the limited medical school options in the country, these medical schools are highly competitive, both with high national exam cutoff score requirements and limited intake capacity. Although females represented 45.2% of higher education enrollment in the 2017-18 academic year and 55.1% of science combinations, the ratio of female to male medical students in Rwanda was 1 to 2.3 [2, 13, 14]. In line with this, this study aims to explore the support systems serving as enablers or barriers to female students pursuing medical school in Rwanda and to recommend ways to increase female participation through support systems.

## Methods

### Design

This study uses a qualitative interpretive methodology that aims to explore the gender-based assumptions and practices within the selected target communities and interpret what factors influence female students’ participation in MBBS programs. The study follows the use of grounded theory, among the interpretive methodologies, to identify ways to strengthen female student enrollment in MBBS programs. In alignment with this, qualitative methods were employed to allow for greater exploration into the contextually relevant, gender-based deterrents to pursuing an MBBS.

### Study setting

This is a cross-sectional study whereby the data was collected from students and gatekeepers, including teachers and parents/guardians at secondary schools in Rwanda. Furthermore, the study setting also included students from three universities in Rwanda that offer an MBBS degree program.

### Sampling and recruitment

#### School selection

According to *The Fifth Integrated Household Living Survey* (EICV5) by the National Institute of Statistics of Rwanda (NISR)’s measurement of the socio-economic trends on a national level, Kigali was ranked as the most resourced province of Rwanda, which attests to high educational capacity, whereas the Eastern province was ranked as the least resourced province of Rwanda. Therefore, in the efforts to represent best and least resourced schools, this study was conducted in sample secondary schools in these two provinces of Rwanda, including Kigali and the Eastern Province. Based on the sampling strategy defined and using the ranking of secondary schools by the Ministry of Education and Rwanda Education Bureau (REB) in 2018, fifteen schools that fit the criteria were identified [15].

Of the fifteen schools selected, four schools could not be considered due to either shutting down, or a lack of interest in participating. Two out of these four were replaced with the next most fitting school according to the criteria. The remaining two could not be replaced without compromising the selection criteria. The thirteen schools that participated in the study are outlined in Table 1:

**Table 1:** Summary of selected secondary schools

Province & Gender Mix	Private	Public
**Kigali – Coed**	APACE Secondary School King David Academy	Lycée de Kigali Rugunga Secondary School
**Kigali – Girls Only**	FAWE Girls School	Lycée Notre Dame de Citeaux
**East – Coed**	Institut Don Bosco Kabarondo Gahini Secondary School	GS Kabarondo B Agape Musha Secondary School
**East – Girls Only**	Gashora Girls School Maryhills Girls Academy	New Explorers Girls Academy

The selection criteria for the universities were done based solely on one criterion, which was having an MBBS degree offering, regardless of the socio-economic status of the geographic location of the university. Therefore, three universities were sampled, including the University of Global Health Equity (UGHE), the Adventist University of Central Africa (AUCA), and the University of Rwanda (UR).

Once the schools were identified, the school administrators, together with the data collectors, identified participants based on the categories in the following inclusion criteria. Participant categories included the different stakeholders, including students, parents, and teachers, as well as the sex composition in each category (e.g., male, female, co-ed).

### Data collection

Data was collected via semi-structured focus group discussions (FGDs), with each FGD having 8–10 participants. The FGD tool was developed based on gender-based analysis frameworks that examined norms, control over resources and benefits, access to education, division of labor within the target communities, and influencing factors such as gender, social, economic, and other constraints to females’ participation in MBBS programs. The FGD tool was piloted and refined before data collection.

Specifically, data was collected through single-sex FGDs with female and male students in science combinations in grades 10–12; single-sex FGDs with male and female students enrolled in an MBBS program; individual interviews with male and female parents of female students in both science and non-science combinations; and co-ed FGDs with secondary school teachers in both science and non-science combinations. Overall, a total of 34 FGDs were conducted (e.g., 28 secondary-level and 6 university-level) and 16 individual interviews.

### Data analysis

The FGDs were audio recorded, translated, and transcribed. The transcripts were coded inductively, with each transcript being blind coded by at least two researchers and then reviewed collectively by three researchers across all transcripts to ensure consistency in the coding process. Following the initial coding process, common themes and patterns were identified.

## Results

Participants highlighted surrounding support systems that have the potential to serve as either enablers or barriers to females from pursuing an MBBS. While participants were asked questions related to joining medical school, secondary school student and parent responses were largely linked to pursuing science-related secondary combinations, which is a pre-requisite for applying to medical school. Therefore, the support systems highlighted as enablers or barriers to females to pursue an MBBS are also linked to joining science combinations as the pre-requisite. Four overarching support systems impacting females’ decisions to pursue an MBBS program emerged from the data, including teacher support, parental and familial support, financial and institutional policy support, and having access to mentors or role models.

### Teacher support

Participants highlighted teacher support as an enabler to females pursuing an MBBS, especially when those teachers instill a love of science in female students and encourage them to pursue science-related fields. Participants indicated that this support could come at different educational levels and should start with primary-level students. For example, one secondary student recalled a Kinyarwanda proverb about correcting a tree early on, linking it to instilling a love of science in girls:

*There is this saying in Kinyarwanda that the correction of a tree is done at its younger stage, so if the government wants girls to have a dream of becoming doctors, to find themselves in the sciences without being afraid of them and having self-confidence, then they have to start from primary schools and encourage them to love studying, especially science subjects.* (Female Secondary Student).

A female medical student also indicated that O-level teachers can instill this love of sciences in students early, so they pursue science tracks, making them eligible to pursue medical school:

*Apart from supporting you and helping you to understand in that subject, they also inspire you to continue, because from the support you get from them, you really see that you are able to do so.* (Female medical student)

Participants highlighted that feedback from teachers and the career directions they encourage students to go into has an impact on student career selection. Furthermore, when teachers encourage students to go into other fields, it can serve as a deterrent to pursuing medicine. One secondary student indicated that teachers seem to be encouraging students to pursue vocational school, rather than the sciences or medicine:

*What I think the school can do… tell us its importance, its advantages, so as a role, teachers are the ones who play the biggest role for us to finish O-level and we choose science courses in A-level… now days they encourage us to study TVET [vocational school], because they tell us since you studied this, you will do this and this.* (Female Secondary Student).

### Parental and familial support

Participants indicated that parental and familial support can serve as an enabler to female students pursuing medicine. For example, female medical students reflected on how culture and encouragement from parents and families impacts their career selection:

*We cannot also forget to talk about our culture in Rwanda. You sometimes find that our choices are affected by the families that we are born in and the culture we were taught… They may also grow up looking at their sisters, hearing from what their parents tell them as Rwandans, then this also affects their choices.* (Female medical student).

*It’s so challenging to pursue any career, not only medicine, if you don’t have the support of parents… specifically for medicine, since it is emotionally and mentally draining: so much cost, so much time…so I think it is always needed for support from the family membesr to pursue any career, specifically [they] are very needed to pursue medicine.* (Female medical student)

A female parent echoed this notion, calling on parents to encourage their daughters to love science:

*You should also encourage them [girls] to love sciences, and for parents, let’s go ahead and encourage our daughters and let them love sciences.* (Female Parent).

Additionally, another secondary student indicated that parents who understand the benefits of pursuing science have an influence over their children:

*The way I see it, some go to study sciences because of parents’ influence, because there are some parents who know the benefits of studying sciences and convince them to study sciences.* (Male Secondary Student).

Alongside being an enabler to pursuing medicine, participants also highlighted that parental support may help female students to endure difficult science courses and to persevere towards the career:

*I think parents’ support is especially important. For me, growing [up] with my daddy, he was always like you are going to take math… But also, as they support me, it’s that support which is necessary because that support keeps me moving at a time, and I say you know what, let me pursue what I want to do.* (Female Secondary Student).

One secondary student echoed this, saying that her parents’ support encouraged her to persevere through difficult subjects and kept her accountable to continue with it:

*Parents’ support is needed, like there was a time when I was about to give up on maths. Seriously it was really hard for me, and sometimes you could hear your friends saying that it is really hard that they are going to drop it, and they also start asking you what are you still doing in maths?…I think listening to parents is necessary, because they have gone through so much, they have seen so much…So it gives me more reasons to work hard and do your best.* (Female Secondary Student).

In addition to parental support being an enabler for girls pursuing medicine, one teacher explained that this support is both linked to the status parents hold in the community, as well as parents recognizing the prestige that comes with a career in medicine. Parents understanding this prestige and impact of the career can enable female students to pursue an MBBS.

*In my point of view, parents support their children to go to study medicine… because that is big status in the community… But for that, you ask if parents support girls, they do support them, because a parents feel proud of having a child who studied medicine; working in a hospital helping people to come back in life. So, parents support them because status is well respected in the society.* (Mixed-sex FGD of teachers).

### Financial and institutional policy support

Participants indicated that other special financial incentives and other policy support systems may enable females to pursue an MBBS. Specifically, institutional or government support were identified as an enabler to pursuing an MBBS, including awareness campaigns, the involvement of relevant government entities, affirmative action policies, and scholarship for girls. With regards to high-level awareness campaigns involving national entities, one parent indicated that the sector leadership can encourage girls to pursue the sciences and medicine in their respective areas:

*The sector leadership where the school is located, in collaboration with the chiefs of education at the sector level, they should encourage girl children to like sciences combinations. Another thing they can do is to provide an incentive to motivate those girls who have already joined science combinations, which would attract other female children to also consider studying science combinations.* (Male Parent).

A secondary teacher echoed, suggesting the development of a strategy to advocate for girls pursuing medicine, especially in more rural areas:

*I think that organizations that do advocacy for girls and MIGEPROF* [Ministry of Gender and Family Promotion] *can establish special ways to help girls, because girls face a lot of challenges in their studies especially in rural areas. Special preparation of girls that I am saying is that they should go down there in the village and take those intelligent girls, and gather them together in the holidays and give them more information about medicine…I think that those girls from rural areas need special treatment because they are intelligent, but the struggles they live with don’t allow them to excel very well. *(Secondary Teacher).

Alongside campaigns directed towards girls from rural areas, participants highlighted that national awareness campaigns may enable students from all backgrounds and regions of the country to pursue medicine through increased awareness of medicine as a career choice:

*Many medical colleges have access to this [admissions] information, but rural children do not know it. So, increase in awareness for everyone, wherever they are; would also help to increase the number of girls in medical school.* (Mixed-sex FGD of teachers).

As an additional institutional support system to access an MBBS, some participants indicated that affirmative action strategies or other adjustments for entry into medical degree programs may also be an enabler to females pursuing an MBBS:

*What I have seen that was done is that in O-Level the required marks for girls to go in A-Level was a little bit less than what was required for boys so that they can favor them and study it. That’s what I have seen.* (Male Medical Student).

One teacher noted that while there are secondary girls schools focused on the sciences, there is a gap in them joining medicine, which could be filled through these policies:

*When you look in single schools for girls you will see they are all in sciences…but in the university level we do not have girls’ doctors. This means that they do not continue to be told the benefits of medicine, or the benefits of surgery. So, it means that we need mobilization for S6 [Senior 6] sciences [combinations].* (Mixed-sex FGD of teachers).

Additionally, scholarships and other financial incentives were highlighted as an enabler for female students from lower socioeconomic backgrounds:

*In the University of Rwanda, most of the time they give scholarships, but we don’t know if they consider gender so that they could favor girls. But whatever the case, the girls in medical sciences are still very few… When a person wants to go for medicine you will see them going outside of our country just because you can’t get it into private institutions, and you will find that because of the financial status of your parents, you decide to go for other courses.* (Female medical student)

### Mentorship and role models

Having access to mentors, role models, and people to look up to was highlighted as another gender-based support system towards pursuing an MBBS. Specifically, participants highlighted that female students may be more likely to go into medicine if they have female doctors to look up to and learn from.

*They [female science teachers] are good examples to other girls and make them understand that they don’t have to be afraid to study science when there are fellow women who teach science.* (Mixed-sex FGD of teachers).

Career day was highlighted as a potential channel to bring female role models in medicine to encourage students to pursue medicine:

*On a career day, they bring these women of motivation to come to these students and tell them how they faced those challenges towards their careers and give them proof of what sciences have done…if you bring someone who has the proof of what sciences have done it will change many opinions.* (Female Secondary Student).

Female secondary students also indicated that having mentors and role models will allow them to see the impact that joining medicine will have on their lives and careers:

*In my understanding, in school there should be discussions with girls who are in sciences somewhere, and schools should bring motivational speakers for girls to encourage them to join sciences.* (Female Secondary Student)

*They have to bring our role models near us so that we see them and talk to them, so that they can encourage us to join sciences.* (Female Secondary Student).

## Discussion

The findings of this study suggest that the surrounding support systems can either enable or serve as barriers to female students pursuing an MBBS. Specifically, strong support systems may have a positive influence over a student’s career selection and subsequent perseverance while pursuing medicine. Additionally, institutional-level policies and female mentorship programs may serve as facilitators to female students pursuing an MBBS. Studies argue that institutions play a key foundation role in implementing and being accountable for policies and systems to promote gender equity [6]. While Rwanda has national-level policies and guidelines for this, they are not fully implemented at the medical school level, meaning that while significant progress is seen in gender representation at the primary and secondary level, there is still a gap at in overall medical school gender representation.

### Influence of support systems on career selection and perseverance

The findings suggest that support from teachers and parents can not only instill a love of sciences in girls but also increase their perseverance towards pursuing it. Selecting a career trajectory is one of a student’s most significant life decisions, so it is critical to understand the barriers and facilitators towards selecting a particular career path and whether these barriers vary by gender. Additionally, beyond just exam scores, career choice is influenced by several factors, such as family and community expectations, socioeconomic status, and individual perceptions regarding career paths [16]. From a gender perspective, studies have shown that female students often make personal career decisions not only on an individual level, but also considering other contextual factors [17]. In line with the findings, other studies in SSA have found that family members play a significant and influential role in students pursuing medicine, regardless of gender [18].

From a social learning theory perspective, environmental forces and influences have a significant impact on career choice and also influence a student’s perseverance when they face challenges in pursuing a particular career [19]. This influence may be further exacerbated by the female students themselves, especially if there is a perceived tension between pursuing medicine and aligning with their perceived gender role expectations. In addition to instilling a love of sciences and increasing female student perseverance, community awareness campaigns about women in medicine may not only facilitate girls to pursue medicine but may also increase the awareness of their parents, communities, and teachers in supporting them. These campaigns may be effective if they address and challenge traditional gender roles as they relate to career selection, as girls are more likely to persevere when these traditional gender roles are challenged [20].

### Female mentorship programs as an enabler to access

The strategic importance of mentorship in academic institutions should be recognized by academic leaders, schools, and faculty at various levels, since leveraging of human resource is an impactful and cost-effective method to ensuring students’ success [21]. Beyond secondary level education, mentorship is crucial for medical school students, especially those pursuing research roles. To ensure improved representation of females in higher level education, increased enrollment and improved performance levels, there is a need to provide guidance in secondary level education. In this regard, the findings of this study highlight the key role of mentors and role models, especially that of teachers, medical professionals, and student peers, in encouraging females to pursue MBBS in Rwanda.

In the case of Rwanda, the perception and role of stakeholders, such as teachers, peers, family, and community are central to females’ access to the required mentorship. In support of this, some study participants highlighted how female students are more likely to go into medicine if they have female role models or female doctors to look up to. However, given the presence of fewer number of female doctors in Rwanda, and how those are also not easily accessible to all girls, especially in rural areas, many female students may not get firsthand experience to interact with and learn from such role models and mentors. Therefore, the difference in accessibility of mentorship for female and male students starts with the lower representation of females in STEM and MBBS fields.

On the other hand, some of the mentioned activities, including the engagement of female teachers in science tracks and organization of career days in secondary schools in some of the sample schools could be replicated across all schools in Rwanda. Engaging female mentors from STEM backgrounds for younger girls could be taken as a critical step in improving females’ engagement in STEM fields and subsequently in MBBS.

As Rwanda has shown commitment to promoting girls’ enrollment through the *Girls’ Education Policy* and indicated existing gender disparities in STEM fields, this commitment could be further supported through national mentorship programs that demonstrate success stories of female doctors and promote women towards going into the medical field. This is especially relevant for girls in rural areas that have limited access to role models within local communities.

### Institutional-level support systems

Nearly all the institutional-level support systems highlighted by participants as having a positive influence over female students pursuing an MBBS can be found in the *Girls’ Education Policy.* These systems include affirmative action policies, like lower cut-off grades for girls, scholarships for female students and advocacy or awareness-raising campaigns to challenge community and familial stereotypes and biases towards girls in sciences. The policy was astute in its strategies, and fifteen years later, various research on increasing girls’ participation and perseverance in sciences further agrees with it. A specialized approach might resolve issues such as the fact that while there is a higher number of girls being promoted from primary to secondary, and attending secondary school, more of them are repeating grades and dropping out of school during the transition from lower to upper secondary [22]. The female science student’s majority in secondary school is also not yet reflected at the university level, and neither the NISR nor MINEDUC’s Statistical Yearbooks provide gender-disaggregated data on medical students, surgical residents, or health workers in Rwanda. However, while the affirmative action policies are found in the national guidelines and policies, these are not fully implemented across all medical schools to ensure equitable gender representation.

Besides the institutional-level support systems highlighted by the study participants, there are other strategies that were included in the 2008 policy and have more recently been recommended by the Gender Monitoring Office that would work to mitigate the mentioned challenges. These strategies look at increasing female science teachers, introducing gender-responsive pedagogy, along with career guidance for female students from primary through university level and incentives/awards for girl learners in science. According to one GMO report, accelerating the approval and publication of the revised policy is essential to achieving these goals, supported through a proper implementation and follow-up process of its strategies. In terms of financial support, mandating a quota for national and international scholarships for girls, who as of 2019–2020 only respectively made up 34.2% and 32% of recipients, would be far-reaching [23].

As part of the follow-up plan, creating a committee to oversee the monitoring and evaluation of gender in the STEM and medical fields through the implementation of these support systems may better guarantee the *Girls’ Education Policy* implementation and success. Inter-agencies’ work might also amplify these institutional-level support systems and provide a wholistic approach at tackling these issues that prevent girls’ participation and perseverance in school.

## Conclusion

This study provides the perceptions of and recommendations from secondary and medical students, parents/guardians, and teachers on the impact of support systems as facilitators or barriers to female students pursuing an MBBS in Rwanda. Specifically, the findings from this study suggest that parental and teacher support, financial and institutional policy support, as well as mentorship and role modeling structures may enable more female students towards pursuing an MBBS. While these provide useful baseline perceptions to inform targeted interventions, further research should be conducted on the effectiveness of their implementation and the overall impact of these recommendations within specific contexts over time.

## Data Availability

The datasets used and/or analyzed during the current study are available from the corresponding author on reasonable request.
